# The association of maternal dietary quality and the antioxidant-proxidant balance of human milk

**DOI:** 10.1186/s13006-022-00498-1

**Published:** 2022-08-08

**Authors:** Samira Karbasi, Afsane Bahrami, Zahra Asadi, Fatemeh Shahbeiki, Mohsen Naseri, Asghar Zarban, Gordon A. Ferns

**Affiliations:** 1 Department of Molecular Medicine, School of Medicine, Cardiovascular Diseases Research Center, Birjand University of Medical Sciences, Birjand, Iran; 2 Clinical Research Development Unit of Akbar Hospital, Mashhad University of Medical Sciences, Mashhad, Iran; 3 Clinical Research Unit, Imam Reza Hospital, Faculty of Medicine, Mashhad University of Medical Sciences, Mashhad, Iran; 4 Metabolic Syndrome Research Center, Mashhad University of Medical Sciences, Mashhad, Iran; 5 Laboratory Technician in the Central Laboratory of Mashhad University of Medical Sciences, Mashhad, Iran; 6 Cellular and Molecular Research Center, Birjand University of Medical Sciences, Birjand, Iran; 7 Cardiovascular Diseases Research Center, Birjand University of Medical Sciences, Birjand, Iran; 8 Clinical Biochemistry Department, Faculty of Medicine, Birjand University of Medical Sciences, Birjand, Iran; 9 Brighton & Sussex Medical School, Division of Medical Education, Falmer, Brighton, Sussex, BN1 9PH UK

**Keywords:** Oxidant-antioxidant balance, Dietary pattern, Breastfeeding mothers, Human milk, Infant

## Abstract

**Background:**

Human milk composition varies over time within an individual mother as well as between lactating mothers due to several factors including maternal health, diet, and nutritional status. Therefore, improving nutrition status during gestation and breastfeeding is crucial for improving the health of both mothers and infants. Diet can enhance the oxidant-antioxidant balance of human milk. This study aimed to investigate the association between human milk oxidant-antioxidant balance with dietary patterns of lactating mothers identified by using principal component analysis.

**Method:**

This cross-sectional study included 350 breastfeeding women between the ages of 20 to 35 years. The dietary intakes of the women in the study were estimated using a validated food frequency questionnaire, which included 65 food items. The oxidant-antioxidant balance of milk samples was assessed using the ferric reducing antioxidant power (FRAP), 2, 2′-diphenyl-1-picrylhydrazyl (DPPH), thiobarbituric acid reactive substances (TBARs), and Ellman’s assay. The milk concentration of total protein, calcium, and triglyceride was also measured using commercial kits.

**Result:**

Two predominant dietary patterns were recognized that we defined as a healthy and unhealthy pattern. There were higher levels of DPPH and thiol in the milk from mothers in the third tertile (highest adherence) of a healthy dietary pattern compared to the first tertile (lowest adherence; *p* < 0.05). Milk calcium and thiol were significantly lower in the third tertile of mothers with an unhealthy dietary pattern versus the first tertile (*P* < 0.05). In multivariate multinomial logistic regression analyses adjusted for mother’s age, body mass index (BMI), energy intake, and infant’s sex, adherence to a healthy dietary pattern was associated with higher levels of milk DPPH (OR = 1.32, 95% confidence interval (CI): 1.01, 1.80) and milk thiol (OR = 1.21, 95% CI: 1.10, 1.50). On the other adherence to the unhealthy dietary pattern was correlated with low levels of milk thiol (OR = 1.29; 95%CI: 1.09, 1.59) and milk calcium (OR = 1.28; 95%CI: 1.11, 1.55).

**Conclusion:**

Our findings demonstrated that adherence to a healthy dietary pattern, identified by higher consumption of green vegetables, other vegetables, and fruits is associated with a higher milk oxidant-antioxidant status in breastfeeding mothers.

## Introduction

Human milk is the best nutrition for infants [1]. It is a natural food containing all of the necessary nutritional components, fluids, and energy they need. Not only is it beneficial for the newborn’s physical growth and development, but also it is beneficial for the psychological and social facets of the development of both infants and mothers [2]. Compared to infant milk formula that has a standardized composition, human milk composition varies dynamically during a feeding, by the time of day, over the period of lactation, and between women and populations. It is affected by genetic, demographic, and environmental variables, infant sex and infectious status, as well as by the mother’s lifestyle, especially dietary habits [3]. In addition to having a high nutritional value and enhancing immunological function, human milk has important anti-oxidant properties that may be useful for infants [4]. A high intake of dairy products, fruit and vegetables, grains, and nuts has been observed to raise the total antioxidant activity of human milk [5]. A mother’s dietary consumption of antioxidants can reduce the risk of oxidative stress and protect against broad-spectrum illnesses in her infant [6]. The mother’s diet can also influence the composition of human milk [7]. The effect of maternal dietary patterns on human milk composition depends on the amount of different nutrients mothers consumed during this period. The concentrations of essential fatty acids, water-soluble and fat-soluble vitamins in human milk are usually dependent on the amount of dietary consumption of these nutrients by lactating mothers [8]. Although protein concentration of human milk can differ between lactating mothers with different dietary habits, it has been shown to be insignificant. Having a healthy dietary habit during and after gestation is necessary to achieve optimal maternal and infant health [9].

There are several limitations of previous research that have focused on the association between breastfeeding mothers’ diet and the oxidant-antioxidant balance of human milk including a limited sample size of studies. This study was performed to assess the association between the oxidant-antioxidant balance of human milk with components of major dietary patterns.

## Methods

### Study setting

This cross-sectional study was performed at Birjand University of Medical Sciences on a sample of 350 mothers who were recruited between January–February 2021 from 4 health centers in different regions of Birjand, South Khorasan, Iran. Inclusion criteria included; healthy women between the ages of 20–35 years, with an infant 1–6 months old. We did not include mothers with any acute or chronic illnesses. Mothers in this survey participated voluntarily and all of them gave written informed consent. Demographic, anthropometric, psychological, and dietary pattern information was determined by the means of a questionnaire. The sample size for the study was calculated according to 80% power and the following formula: n = [(Z_1-α/2_)^2^ -S^2^]/d^2^ which SD = 5.2, d = 0.504, and α = 0.1 [10]. According to this calculation, 294 participants were required; nevertheless, 350 participants were added due to data availability and to account for every possible exclusion. The study protocol was approved by the Ethics Committee of Birjand University of Medical Sciences.

### Demographic and anthropometric variables evaluation

A trained nurse evaluated information including mother age, systolic blood pressure (SBP), type of delivery, body mass index (BMI), economic status, parent death, parent divorce, father s’ and mother s’ educational attainment, infant age, infant sex and infant head circumference. Participants’ height and weight were measured and BMI was calculated from these values as weight in kilograms divided by height in meters squared. Height and around the infant’s head circumference (cm) was recorded using an inelastic measuring tape to the nearest mm. Electronic scales were used to measure weight to the nearest 0.1 kg. SBP values in individuals in resting conditions were measured and repeated during the same visit [11].

#### Social-economic status and family situation

A standard questionnaire was used to collect data on family factors and socio-economic status. In terms of socio-economic variables, the highest mother’s and father’s education attainments were evaluated separately, with 3 response options: 'elementary (9 years),' 'intermediate (10–12 years),' and 'university (13 years).' Moreover, economic status was assessed by asking mothers to report their economic condition by choosing one of three answer choices: less than enough, enough, or more than enough. In related to family structure, mothers were asked to indicate parent death (Yes or No) and parent divorce (Yes or No).

### Milk sampling

Human milk samples (1 to 6 months postpartum) were collected manually in the morning between 7 and 10 am. Samples were stored in a sterile tube. Before infant feeding, samples were taken of one breast in the morning. Each mother provided two 20 ml human milk samples (for a total of 700 samples). Samples were stored in sterile tubes and were transferred to our laboratory on dry ice. Samples were freeze-dried and then stored at − 80 °C until they were analyzed.

### Oxidant-antioxidant status measurement

#### Ferric reducing, antioxidant power (FRAP) assay

The FRAP assay method was as previously explained by Benzie and Strain; based on the reduction of a Fe3+, tripyridyltriazine complex to the blue-colored ferrous form in the presence of reductants (antioxidants) [12]. Briefly, 10 μL of the test sample, standard (FeSO4) or blank (for each milk sample, a blank sample was used to remove milk turbidity) was mixed with 250 μL of FRAP reagent, freshly prepared (10 volumes of 300 mM acetate buffer, plus1 volume of 10 mM TPTZ solution in 40 mM HCl, plus 1 volume of 20 mM FeCl3· 6H2O) incubated for 10 minutes at 37 °C. The absorbance was measured at 593 nm. All tests were performed in duplicate and the results were described in μmol/L.

#### A-diphenyl- B-Picrylhydrazyl (DPPH) assay

The antioxidant activity of human milk samples was assessed in terms of free radical scavenging activity, according to the Brand-Williams method with a slight modification [13]. Briefly, 50 μl of each human milk and control sample were mixed with 950 μl of the DPPH solution in a test tube. After a 10-minute incubation at 37 °C, it was centrifuged at 3000 g for 3minutes. The absorbance of the reaction mixtures was measured at 517 nm by a spectrophotometric method, and a methanol solution of DPPH was utilized as a control sample. The percent of antiradical efficiency was assessed by the equation that follows: DPPH radical-scavenging activity (%) = [(absorbance of the control – mean absorbance of the sample)/absorbance of the control] * 100. Each test was carried out in duplicate and the results are expressed in μmol Trolox equivalent /L.

#### Thiobarbituric acid reactive substances (TBARs) assay

The final product of lipid peroxidation, malonyldialdehyde (MDA), was measured using the TBARs assay in human milk samples [14]. Samples (100 μl) were mixed with 1 ml of TBARs reagent (7.5 g trichloroacetic acid, 187 mg TBA, and 6.25 ml of chloridric acid). The mixture was heated for 20 minutes in a boiling water bath. Then 1 ml of N-butanol was used to extract TBARs adducts and the solution was centrifuged for 10 minutes at 1500 g at 4 °C. The fluorescence spectrum of these samples was measured at the excitation (515 nm) and emission (553 nm) wavelengths. The results (μmol TBARs/L) were compared to a standard curve.

#### Ellman assay

Total milk thiol concentration or sulfhydryl groups (T-SH) were assessed using the techniques originally described by Ellman and modified by Hu [15]. TS-H interacts with 5-thio-2-nitrobenzoic acid (DTNB), forming a highly colored anion at a maximum peak of 412 nm. In this assay, an aliquot of 50 μL of fresh milk was homogenized with 1 mL of Tris/EDTA buffer (0.25 mmol/L Tris base, 20 mmol/L EDTA, pH 8), 50 μL aliquot of DTNB stock solution (10 mmol/L in absolute methanol) and 650 μL N-butanol. Each duplicate sample was centrifuged at 3000 g for 5 minutes, then absorbance at 412 nm was measured [16]. The absorbance was measured again after 15 min at room temperature with a DTNB blank (with 50 μL methanol). The concentration of T-SH groups was estimated with reduced glutathione as the T-SH group standard and the data was demonstrated in μmol/L.

#### Milk calcium, protein, and triglyceride

All photometric analysis was evaluated using a plate reader (Epoch™, BioTek, Winooski, VT, USA), at 37 °C. Monochromatic readings were conducted to assess all absorbance data [17]. Calcium was estimated with the Arsenazo III kit (Pars Azmoon, Tehran, Iran) according to the manufacturer’s instructions for use. Calcium makes a compound with Arsenazo III at neutral-pH, and the color severity is proportional to the calcium content in the sample [18]. The absorbance of all samples and blanks was assessed at 660 nm.

The Bradford protein assay was undertaken using 10 μl of human milk sample and 1 ml of color reagent in duplicate. For 30 seconds, the contents were mixed and, after 5 minutes of incubation at room temperature, the absorbance values of all samples and blank (10 μl of each human milk sample and 1 ml of distilled water duplicate) were determined using a microplate reader at 595 nm [19].

Milk triglyceride values were assessed using a Pars Azmoon® kit (Tehran, Iran). It is an assay that employs enzymatic hydrolysis and quantification by measuring absorbance at 546 nm and the data is evaluated in mg/dL. An aliquot of 10 μL of human milk sample (1:10 diluted) duplicate was added with 1 mL of triglyceride reagent, vortex mixed, and incubated at 37 °C for 30 mins. The absorbance of all samples and blanks (1 ml of distilled water and 10 μl samples) was assessed at 546 nm.

#### Nutritional assessment

The dietary intake of all mothers was estimated using a validated food frequency questionnaire. This tool contained 65-items with the frequency of intake (per day, week, month, rarely, and never) and portion size for every food item [20]. Expert nutritionists performed face-to-face interviews with all individuals to complete the questionnaires. The analysis of the nutrient consumption of each person was conducted based on the US Department of Agriculture’s National Nutrient Databank [21]. The dietary nutrient intakes of participants were calculated [21] and then dietary patterns were determined based on 25 predefined food groups (Table 1) conforming to the similarity of food frequency questionnaire food items.

**Table 1:** Food groupings used in factor analysis of dietary patterns

Food group	Food item
Refined grains	White breads, rice, Pasta
Whole grains	Dark breads (Iranian),
Fast foods	Pizza, processed meat
Snacks	Biscuits, cakes and pastries, chocolate, ice-cream, chips
Dairy products	Whole milk, low-fat milk, yogurt, breakfast cheese, Dough
Solid fats	Butter, cream, solid oil, tall, salad dressing
Liquid fats	Liquid oil, olive oil
Sugars	Sugar loaf, diabetic sugar, sugar
Honey	Honey
Fruits	Tree fruit, seasonal fruit, fruit compote, fruit juice, dried fruits
Carbonated beverages	Soft drinks, beer, diet drinks
Tea	Tea
Coffee	Coffee, coffee and milk, Nescafe
Legumes	Beans, soy
Pickled foods	Pickles, salty Cucumber
Green vegetables	The vegetables, lettuce, spinach
Other vegetables	Garlic, onion, tomato, Cucumber, salad (mixed salad of tomato, Cucumber and onion)
Potato	Boiled potato, other potatoes, french fries
Liquid foods	Soup
Eggs	Boiled egg, scrambled eggs
Red Meats	Lamb meat, beef, hunting meat
Organ meat	Heart, liver and kidney, intestine and viscera
Seafood	Fish, fish tuna, shrimp
Chicken	Poultry
Nuts	Walnut, all types of nuts

#### Evaluation of dietary patterns

The process for determining dietary patterns (exposure) from food intake data involved first grouping the 65 food items into the 25 predefined food groups (g day ^− 1^) as predictor variables based on their similarity. We used factor analysis (Principal Component Analysis) to create dietary habits based on 25 foods or food groups [22]. To preserve the factors uncorrelated and facilitate interpretation, they were rotated orthogonally (varimax rotations). Factors with an eigenvalue > 1, the screen test, and the interpretability of the factors were taken into account while choosing the number of factors to keep [23]. As a result, the current study yielded a three-component dietary pattern (tertile). The first component (first tertile) shows how one dietary pattern is consumed less frequently, while the last component (third tertile) shows how it is consumed more frequently. Because it was the most interpretable component in the sample population. The first component of each dietary pattern was selected as the reference group. The factor scores for every dietary pattern were determined by summing the intake of the food groups adjusted by their factor loadings which were obtained by factor loadings. Finally, each mother was assigned a score in each of the two defined dietary patterns. Because the percentage of variance described by each dietary pattern is highly dependent on the number of variables used in the analysis, this criterion was not reported [24]. The reference category for every dietary pattern was selected as the first tertile.

### Statistical analysis

All analyses were conducted using the Statistical Package for the Social Sciences (SPSS) version 16. Statistical data was measured for normality by using the Kolmogorov-Smirnov test. Continuous and categorical indices are displayed by Mean ± SD and number (percent), respectively. The ANOVA test was applied to measure the significant difference in normal variables among groups. Participants in the type of delivery and baby sex across tertiles of dietary patterns were compared by using the chi-square test. Multivariate logistic regression was used to evaluate the associations between a component of the score built to evaluate the adherence to the healthy, unhealthy pattern. All analyses were considered using two-tailed tests and a *P*-value < 0.05 was set as significant.

## Results

### Food groupings used in factor analysis of dietary patterns in Table 1

The dietary patterns of the study population were assessed using principal component analysis; two main dietary patterns were identified, which we labeled as healthy and unhealthy dietary patterns.

### Principal component analysis identified a factor loading matrix for major dietary patterns in Table 2

The factor analysis approach was used to enter data on food intake for the 25 designated food groups. Two major dietary patterns emerged from the scree plot of eigenvalues. The greater the contribution of an item of food or group to a certain component, the higher the loading factor of that item of food or group. The ‘healthy’ dietary pattern was characterized by high consumption of refined grains, legumes, whole grains, other vegetables, eggs, and red meat. The second dietary pattern, labeled as ‘unhealthy’ dietary pattern, was characterized by a high intake of carbonated beverages, snacks, honey, seafood, and chicken.

**Table 2:** Principal component analysis identified a factor loading matrix for major dietary patterns

Food group	Dietary patterns	
	Healthy	Unhealthy
Refined grains	0.61	–
Legumes	0.59	–
Whole grains	0.51	–
Other vegetables	0.50	–
Green vegetables	0.48	
Eggs	0.48	–
Nuts	0.47	
Fruits	0.46	
Red meat	0.40	–
Solid fats	–	–
Tea	–	–
Potato	–	–
Dairy products	–	–
Liquid fat	–	–
Sugars	–	–
Carbonated beverages	–	0.78
Snacks	–	0.63
Honey	–	0.59
Seafood	–	0.53
Chicken	–	0.48
Fast foods		0.45
Pickled foods	–	–
Coffee	–	–
Organ meat	–	–
Liquid foods	–	–

*Values less than 0.40 are not reported

### Demographic, anthropometric, and socio-economic characteristics of participants in different tertiles of obtained dietary patterns in Table 3

The average age of mothers was 29.5 ± 5.9 years. The dietary patterns scores were used to classify the participants into tertiles: T1 (low adherence to the dietary patterns), T2 (moderate adherence to the dietary patterns), and T3 (high adherence to the dietary patterns). There was no significant association between the demographic and socio-economic characteristics of the individuals in different tertiles of identified dietary patterns containing: mother age, SBP, type of delivery, BMI, economic status, parent death, parent divorce, father s’ and mother s’ educational attainment, infant age, infant sex and infant head circumference (*P* > 0.05).

**Table 3:** Demographic, anthropometric and socio-economic characteristics of participants in different tertiles of obtained dietary patterns

Variables (score)		Healthy pattern		*P*-value†	Unhealthy pattern		*P*-value†
		Tertile 1 (*n* = 59)	Tertile 3 (*n* = 60)		Tertile 1 (*n* = 51)	Tertile 3 (*n* = 62)	
Mother Age (y)		29.2 ± 5.7	29.3 ± 6.7	0.65^a^	29.3 ± 5.7	29.4 ± 6.2	0.98^a^
Mother SBP (mmHg)		105 ± 78	102 ± 14	0.75^a^	102 ± 78	100 ± 14	0.11^a^
Type of Delivery (natural), n (%)		37.3%	43.2%	0.22^b^	42.3%	37.1%	0.31^b^
Mother BMI (Kg/m2)		23.5 ± 4.2	25.1 ± 4.1	0.10^a^	23.9 ± 3.2	26.1 ± 4.1	0.14^a^
Infant Age (day)		139 ± 35.8	139.7 ± 36.4	0.46^a^	137.6 ± 56.9	147.6 ± 43.6	0.16^a^
Infant Sex (male), n (%)		33.3%	40.1%	0.36^b^	44.3%	39.1%	0.87^b^
Infant Head Circumference (cm)		38.7 ± 23	39.9 ± 34	0.22^a^	44.0 ± 21	39.7 ± 28	0.10^a^
Economic status	Less than enough	24.5%	25%	0.58^b^	53.1%	35.5%	0.11^b^
	Enough	73.5%	73%	0.59^b^	45.9%	63.5%	0.31^b^
	More than enough	2%	2%	0.91^b^	1%	1%	0.86^b^
Parent death	Yes	1%	4%	0.19^b^	2.1%	7.9%	0.14^b^
	No	99%	96%	0.31^b^	97.9%	92.1%	0.13^b^
Parent divorce	Yes	0%	0%	0.12^b^	0%	2%	0.15^b^
	No	100%	100%	0.10^b^	100%	98%	0.11^b^
Father’s education (year)	0–9	27.5%	28%	0.25^b^	27.5%	14.9%	0.71^b^
	10–12	44.9%	27%	0.09^b^	39.6%	34.8%	0.54^b^
	> 13	27.6%	45%	0.42^b^	32.9%	50.4%	0.32^b^
Mother’s education (year)	0–9	22.4%	25%	0.11^b^	36.4%	10.9%	0.32^b^
	10–12	44.9%	25%	0.23^b^	39.6%	19.8%	0.27^b^
	> 13	32.7%	50%	0.11^b^	24%	69.3%	0.33^b^
Mothers’ chronic disease history	Yes	6.2%	9.1%	0.27^b^	11.5%	10.1%	0.52^b^
	No	93.8%	90.9%	0.28^b^	88.5%	89.9%	0.66^b^

Abbreviations: *BMI* body mass index, *SBP* systolic blood pressure, Data presented as Mean ± SD or frequency (percent)

^a^ *p*-value from analysis of the variance (ANOVA) for groups comparison

^b^ using chi-square test

### Human milk composition by tertiles of identified dietary patterns in Table 4

Participants were divided into 2 groups based on their dietary pattern scores: participants with healthy dietary pattern (*n* = 181) and mothers with unhealthy dietary pattern (*n* = 169). Mothers in the highest tertile of the healthy dietary pattern had higher scores of milk DPPH and thiol, compared to those in the lowest tertile of the healthy dietary pattern (DPPH 347 ± 163 μmol/L vs. 321 ± 94 μmol/L, *P* < 0.034 and thiol 81.3 ± 20.7 μmol/L vs. 73.5 ± 15.6 μmol/L *P* < 0.012). Milk calcium and thiol were significantly lower in the third tertile of the unhealthy dietary pattern versus the first tertile (calcium 8.82 ± 1.09 mg/dL vs. 9.20 ± 1.36 mg/dL, *P* < 0.021 and thiol 73.8 ± 18 μmol/L vs. 78.6 ± 21 μmol/L /L, *P* < 0.003).

**Table 4:** Human milk composition by tertiles of identified dietary patterns

Variables	Healthy pattern		*P*- value†	Unhealthy pattern		*P*- value†
	Tertile 1 (*n* = 59)	Tertile 3 (*n* = 60)		Tertile 1 (*n* = 51)	Tertile 3 (*n* = 62)	
Milk DPPH (μmol Trolox equivalent /L)	321 ± 94	347 ± 163	**0.034**	329 ± 75	352 ± 103	0.23
Milk FRAP (μmol /L)	577 ± 149	534 ± 145	0.14	571 ± 141	537 ± 170	0.24
Milk MDA (μmol TBARs/L)	0.12 ± 0.91	0.11 ± 0.72	0.34	0.13 ± 0.89	0.10 ± 0.79	0.08
Milk Thiol (μmol /L)	73.5 ± 15.6	81.3 ± 20.7	**0.012**	78.6 ± 21	73.8 ± 18	**0.003**
Milk Calcium (mg/dL)	9.13 ± 1.31	8.91 ± 1.02	0.46	9.20 ± 1.36	8.82 ± 1.09	**0.021**
Milk Protein (g/dL)	1.64 ± 1.73	1.45 ± 1.61	0.59	1.56 ± 063	1.63 ± 0.78	0.56
Milk Triglyceride (mg/dL)	3.95 ± 1.05	4.11 ± 1.19	0.63	4.20 ± 1.33	4.48 ± 1.55	0.87

†Obtained from ANOVA test, *DPPH* diphenylpicrylhydrazyl, *FRAP* ferric reducing ability of plasma, *MDA* Malondialdehyde

### Multivariate adjusted odds ratios (95% CIs) for low concentration of human milk antioxidant across tertiles of dietary patterns in Table 5

Multivariate multinomial logistic regression for mothers’ milk antioxidants across tertiles of two dietary patterns is described in the crude model in this table. Adherence to an unhealthy dietary pattern was correlated with low levels of milk thiol (OR = 1.29; 95% confidence interval (CI): 1.09, 1.59; 3th tertile with 1st unhealthy dietary pattern tertile) and milk calcium (OR = 1.28; 95%CI: 1.11, 1.55; third tertiles versus the first unhealthy dietary pattern). We also found a significant positive association between adherence to a healthy dietary pattern and levels of milk thiol (OR = 1.21, 95% CI: 1.10, 1.50; 3th tertile with 1st healthy dietary pattern tertile) and milk DPPH (OR = 1.32, 95% CI: 1.01, 1.80; 3th tertile with 1st healthy dietary pattern tertile).

**Table 5:** Multivariate adjusted odds ratios (95% CIs) for low concentration of human milk anti-oxidant across tertiles of dietary patterns

Variables	Healthy pattern			Unhealthy pattern		
	Tertile 1 (*n* = 59)	Tertile 2 (*n* = 62)	Tertile 3 (*n* = 60)	Tertile 1 (*n* = 51)	Tertile 2 (*n* = 56)	Tertile 3 (*n* = 62)
Milk DPPH (μmol Trolox equivalent /L)	1.0	1.5(1.10, 1.82) *	1.32(1.01, 1.80) *	1.0	–	–
Milk Thiol (μmol/L)	1.0	0.98(0.97, 1.013)	1.21(1.10, 1.50) *	1.0	1.01(0.99, 1.02)	1.29(1.09, 1.59) **
Milk Calcium (mg/dL)	1.0	–	–	1.0	1.12(1.06, 1.18) *	1.28(1.11, 1.55) **

Tertile 1 was considered as reference group. Adjusted for mother’s age, BMI, energy intake, and infant sex

*p < 0.05

**p < 0.01

***p < 0.001

## Discussion

To the best of our knowledge, this is the first study that investigates the relationship between dietary pattern and the content of human milk conducted on breastfeeding mothers. We found a significant difference in milk DPPH, thiol, and calcium in mothers with healthy and unhealthy dietary patterns.

Our results demonstrated significantly higher levels of milk DPPH and thiol, measures of milk quality [25], in mothers with a healthy diet compared to mothers with an unhealthy diet. The content of human milk was affected by several variables, some of which are associated with the mother and some with the infant. Changes in breastfeeding and other parameters affecting the mother’s physiology, anthropometric features, nutritional level, and type of diet are key critical [26]. The antioxidant level of the child is strongly depended on the mother’s antioxidant level throughout pregnancy and is preserved after delivery using the synthesis of endogenous molecules by the infant at the expense of exogenous molecules transmitted via milk [27]. The antioxidant capacity of human milk comprises endogenous and exogenous substances, from vitamins (A, E, and C), to enzymes (glutathione peroxidase, superoxide dismutase), metals (copper, zinc, and selenium) which can contribute to a synergistic manner to neutralize free radicals [4]. The total antioxidant capacity (TAC) of mother’s milk shows the antioxidant components’ presence and activity that protect lipids and proteins from oxidative degradation. Ortega et al. have reported a correlation between antioxidant levels in human milk and dietary intake in gestation [28]. These studies imply the likelihood that human milk may be important to reduce oxidative stress, which may lead to a variety of dysfunctions, and that its antioxidant molecule composition alters during breastfeeding in order to provide the greatest care to the infant [4, 29]. A healthy diet and lifestyle throughout gestation appear to be important for achieving an adequate TAC. One of the most common methods of assessing TAC levels in milk is the DPPH assay [30]. Antioxidants that counteract the damaging effects of free radicals on cells and their links to illnesses continue to stimulate the studies of the antioxidant and antiradical capabilities of components included in different natural products and nutritional supplements [31]. Among the most essential antioxidants are polyphenols, which include phenolic compounds and flavonoids. The phenolic compounds are secondary metabolites that are derived from plants and are powerful natural dietary antioxidants [32]. The dietary intake of antioxidants (e.g., carotenoids, flavonoids, and polyphenols) cannot be produced in the human body and must be obtained from the consumption of fruits, vegetables, and cereals [33]. Also, citrus fruits are high in phytonutrients with an antioxidant function that protects the body from free radicals [34]. Sánchez and colleagues reported that human milk from females with an adherence to a healthy diet such as the Mediterranean included twice as many specific phenolic compounds as baby formulas. These diets are known for their abundance of fresh fruits and vegetables, as well as the use of olive oil instead of hard fat [35, 36]. Antioxidants are chemicals that are required for the preservation of cell homeostasis, and their consumption through the diet has positive effects on human health. Low-molecular-weight thiols are one of the important types of antioxidant compounds [37]. The daily intake of thiols via the diet plays an essential role in fighting free radicals and contributes to disease prevention. Plant foods are the most well-known dietary matrix that are high in thiols, antioxidant components, vitamins and minerals [38]. Also, as mentioned in previous studies, a healthy dietary pattern is rich in fruits and vegetables as opposed to an unhealthy dietary pattern [39, 40]. The antioxidant activity of plant foods contains a number of mechanisms like radical scavenging and singlet oxygen quenching [41].

We found a direct relationship between adherence to unhealthy dietary pattern and low levels of milk calcium. Calcium is one of the macro elements necessary for a infant’s normal growth and development. It, a major component in human milk, is important for bone formation, muscular contraction, nerve impulses transmission, and blood coagulation [42]. In mothers’ milk, calcium is the second most common mineral [26]. Daily meals consisting mainly of milk, meat, fish, eggs, and legumes usually meet maternal needs for zinc, magnesium, copper, iron, and calcium [43]. Bailey reported a concentration of 154 mg/L in milk calcium from New Guinea women with poor dietary intake, compared to levels of 260–340 in other previous investigations. Also, Greer et al. have demonstrated an association between a mother’s calcium intake and human-milk calcium [44].

This study has several strengths. Our sample size was large and was derived from a free-living population sample. Also, our classification of dietary intake is generally accepted, and then we compared the results of the two groups to confirm our conclusion. A major limitation of our study is recall bias, as all of the variables related to the questionnaire were taken by the recall method. However a cross-sectional design and retrospective data cannot assess causality.

## Conclusion

The quality of dietary intake appears to be an important factor affecting the composition of human milk. A dietary pattern of high consumption of vegetables and fruits was correlated with higher levels of oxidant-antioxidant factors in human milk. These results indicate that the dietary patterns of breastfeeding females might affect the mother’s milk macronutrient composition and provide a foundation for better infant health.

## Abbreviations

BMI: Body mass index
CI: Confidence interval
DPPH: A-Diphenyl- B-Picrylhydrazyl
FRAP: Ferric Reducing, Antioxidant Power
MDA: Malondialdehyde
SBP: Systolic blood pressure
SPSS: Statistical Package for the Social Sciences
T-SH: Total sulfhydryl
TAC: Total antioxidant capacity
TBARs: Thiobarbituric acid reactive substances
